# Effect of using magnifying loupe glasses on lymphocele formation and surgical outcomes in gynecologic oncology

**DOI:** 10.4274/tjod.galenos.2019.93467

**Published:** 2020-02-28

**Authors:** Fatih Akkuş, Serhan Can İşcan, Jalal Raoufi, Mehmet Güney, Evrim Erdemoğlu

**Affiliations:** 1 Süleyman Demirel University Faculty of Medicine, Department of Obstetrics and Gynecology, Isparta, Turkey; 2 Süleyman Demirel University Faculty of Medicine, Department of Obstetrics and Gynecology, Division of Gynecologic Oncology, Isparta, Turkey

**Keywords:** Gynecologic oncology, magnifying loupe glasses, lymphocele

## Abstract

**Objective:**

To investigate the effect of using magnifying loupes during surgery on surgical outcomes and lymphocele formation.

**Materials and Methods:**

We prospectively enrolled 36 patients with gynecologic cancer who underwent pelvic and para-aortic lymphadenectomy. Age, body mass index, menopausal status, type of cancer, comorbid diseases, preoperative albumin and albumin replacement therapy, performance status, serum CA125, hemoglobin, platelets and white blood cells, surgical procedure, blood loss, blood transfusion, the count of removed lymph nodes, presence of metastatic lymph nodes, total amount of drainage, postoperative complications, operation length, and count of used hemoclips were recorded. Patients were randomized into two groups: group 1 operated using loupe glasses, and group 2, without loupes.

**Results:**

In the loupe-negative group, total drainage volume was 6698 mL, whereas in the loupe-positive group, it was only 1049 mL (p<0.01). Postoperatively, the mean drainage duration was 10.6±5.1 days in loupe-negative group and 4.8±2.4 days in the loupe-positive group (p=0.0001). There were no differences between the two groups in terms of surgical site infections, fascial defects, and pulmonary thromboembolism (p=0.39, 0.33, 0.59, respectively). There was no significant difference in the number of harvested lymph nodes in patients who underwent surgery with or without loupes being used. The count of used hemoclips were 50.22±8.05 and 41.38±9.7 for the loupe-negative and positive groups, respectively (p<0.01). There was no lymphocele in the loupe-positive group, but we detected 5 (27.8%) lymphocele in the loupe-negative group (p=0.05).

**Conclusion:**

Gynecologic oncologic surgeons can add magnifying loupe glasses to their armament and benefit from this technical device; lymphocele development, total drainage volume, length of drainage time, and clip counts can be decreased by using loupe glasses in gynecologic cancer surgery.

**PRECIS:** In gynecologic cancer surgeries, using loupe glasses is beneficial in decreasing lymphocele formation, amount of lymphatic drainage, and hemoclip use.

## Introduction

Pelvic and para-aortic lymphadenectomy is a common procedure in gynecologic oncologic surgery to determine lymph node status and disease stage. Hemorrhage, hematoma, nerve and ureteral injury, postoperative ileus and lymphocele formation due to disruption of lymphatic drainage are well-known complications of lymphadenectomy. Usually, lymphoceles appear within 2 months after surgery and they are mostly asymptomatic. However, they may rarely affect the ureter and bladder (hydronephrosis, urinary frequency), bowel (ileus, tenesmus), or vessels (thrombosis) and cause abdominal pain. Lymphoceles may also become infected^(1,2)^. The incidence of lymphoceles after gynecologic cancer surgery is reported as 1-58%. There are many factors that affect the formation of lymphoceles, the count of removed lymph nodes, the extent of lymphadenectomy, status of ligated lymph vessels, use of retroperitoneal suction drainage, type of cancer, and administration of heparin for thromboembolic prophylaxis^(2,3)^. The success of any techniques has not been proven in the literature and there are a few reports that found a significant reduction in postoperative lymphocele formation after lymphadenectomy^(4)^. Loupes allow to see 2-4 times more magnification than eyes. Loupes have many benefits: wider and deeper field of view, cost, portability. Loupes are used in plastic, maxillofacial, otorhinolaryngologic, ophthalmic, cardiothoracic, and pediatric surgery^(5)^. The use of loupes in gynecologic oncology has been described in nerve-sparing radical hysterectomy^(6)^.

The aim of this study was to investigate the effect of using magnifying loupes during surgery on surgical outcomes and lymphocele formation.

## Materials and Methods

We prospectively enrolled 36 patients with gynecologic cancer who underwent pelvic and para-aortic lymphadenectomy between February 2016 and July 2017 at the Department of Gynecologic Oncology of Süleyman Demirel University. Age, body mass index (BMI), menopausal status, type of cancer, comorbid diseases, preoperative albumin and albumin replacement therapy, performance status [Eastern Co-operative Oncology Group (ECOG) and Karnosky], serum carcinoma antigen 125 (CA125), serum hemoglobin, platelets and white blood cells (WBC), surgical procedure, blood loss, blood transfusion, the count of removed lymph nodes, presence of metastatic lymph nodes, total amount of drainage, postoperative complications, the length of total surgical time, and count of hemoclips used were recorded.

**Ethics Committee Approval:** The study was approved by Local Ethics Committee of Süleyman Demirel University (approval number: 78, date: 20,07, 2016).

### Statistical Analysis

Statistical analysis was performed using MedCalc. The significance level was set as p<0.05. Descriptive analysis was performed using the independent t-test, Mann-Whitney U, and chi-square test. These parameters were evaluated via correlation tests, and multiple regression analysis was performed for efficient parameters. Prediction of the results was evaluated using receiver operating characteristic curve analysis. Power analysis was calculated with 80% power, 5% alpha to a 50% decrease in the total amount of drainage. Patients were randomized into two groups; group 1 operated with the use of loupes and group 2, without loupes.

## Results

Eighteen of 36 patients underwent surgery for endometrial cancer. Fifteen of 36 patients and 3 of 36 patients underwent surgery for ovarian cancer and cervical cancer, respectively. The demographic and surgical data of patients who underwent surgery with and without loupe magnification being used were compared. There were no statistically significant differences in age, BMI, menopausal status, and types of cancer. Additionally, we found no statistically significant difference in laboratory findings (preoperative albumin, preoperative hemoglobin, WBC, platelet counts, and CA125 levels) between the two groups (Table 1). The findings showed no statistically significant differences in surgical procedures (simple hysterectomy, radical hysterectomy, appendectomy, peritonectomy, panniculectomy, urinary and gastrointestinal system surgery), blood transfusions, fresh frozen plasma transfusions, and intraoperative blood loss (Table 2).

However, surgery with loupes decreased the count of hemoclips required to obliterate vessels. The counts of used hemoclips were 50.22±8.05 and 41.38±9.7 for the loupe-negative and positive groups, respectively (p=0.006). Furthermore, the use of loupes decreased the duration of surgery, but this finding was not statistically significant (5.3±1.4 h in the loupe-negative group, and 4.6±1.05 h in the loupe-positive group).

There was no significant difference in the number of harvested lymph nodes in patients who underwent surgery with or without loupes being used. The mean count of dissected pelvic lymph nodes was 20.0 in the loupe-positive group, and 26.5 in the loupe-negative group (p=0.05). The mean count of dissected para-aortic lymph nodes in the loupe-positive group (n=13.5) was more than in the loupe-negative group (n=12) (p=0.93).

Although the amount of drainage after the first and 6 hours of surgery were similar, there was a statistically significant difference after the first day, second day, and total drainage volume. In the loupe-negative group, the mean drainage volume was 454 mL for the postoperative first day, 661 mL for postoperative second day, and 6698 mL for total drainage volume, whereas in the loupe-positive group was 245 mL, 229 mL, and 1049 mL, respectively. Postoperatively, the mean duration of drainage was 10.6±5.1 days in loupe-negative group and 4.8±2.4 days in loupe-positive group (p=0.0001).There were no differences between the two groups in terms of surgical site infection, fascial defect, and pulmonary thromboembolism (p=0.39, 0.33, and 0.59, respectively). Lymphocele formation was lesser in the loupe-positive group than in the other group; there was no lymphocele in the loupe-positive group, but we detected 5 (27.8%) lymphoceles in the loupe-negative group (p=0.05). Multiple regression analysis revealed a significant relationship between total drainage volume, use of loupes, BMI, hemoglobin level, count of dissected pelvic lymph nodes, metastatic pelvic lymph nodes, metastatic para-aortic lymph nodes, surgical time, type of cancer, and ECOG score (Table 3).

## Discussion

This is the first study to analyze the effect of wearing loupes on lymphadenectomy, lymphocele formation, and surgical outcomes in gynecologic oncology surgery. In our study, loupe use reduced surgical time but there was no statistically significance (5.3±1.4 hours in loupe-negative group, and 4.6±1.05 hours in loupe-positive group). We found that the use of loupes was an effective method to reduce the amount of drainage (6698.33±5552.22 mL in loupe-negative group, and 1049.44±943.68 mL in the loupe-positive group, (p=0.002), consequently, reducing postoperative albumin replacement in the loupe-positive group (p=02).Lymph node dissection is the most common etiology of lymphocele and lymphatic leakage^(2,7)^. Previous studies have reported different results about lymphocele incidence. According to these studies, in patients who undergo gynecologic cancer surgery, the incidence of lymphocele is 1-58%. This wide range may be due to different types of cancer, the presence of symptoms, differences between medical centers, differences between types of surgical techniques and intraoperative procedures, and finally different methods for diagnosis. In our study, the lymphocele rate was 27.5% in the loupe-negative group, whereas no lymphocele was found in the loupe-positive group (p=0.05). In a study by Zikan et al.^(7)^ the highest risk of lymphocele formation was reported to be in ovarian cancer, whereas the highest rate was reported in cervical cancer by Kim et al.^(2)^. Another study reported no association between lymphocele formation and cancer type^(8)^. Our study showed no significant difference between the lymphocele rate and type of gynecologic cancer. However, there are no studies about the relationship between the amount of lymphatic drainage and type of cancer. We found that there was more lymphatic drainage in cervical cancer (p<0.05). However, the number of patients included in our study is the main limitation to analyze lymphocele formation in different gynecologic cancers. Gallotta et al.^(8)^ found that the use of laparoscopic clips reduced lymphocele rates in gynecologic cancer surgeries. Magnification with loupes may help surgeons to correctly identify lymphatics and vessels. Therefore, it may decrease the total number of clips required and decrease the total lymphatic drainage and lymphocele formation.

There are some studies about the relationship between lymphatic drainage volume and the count of dissected lymph nodes^(10)^. According to several studies, for endometrial and cervical cancers, and for ovarian cancers, 11 and 20 lymph nodes, respectively, were accepted as sufficient and effective in pelvic lymphadenectomy^(11,12,13)^. There are no data about the maximum count of dissected lymph nodes in gynecologic cancers. In our study, the mean count of dissected pelvic lymph nodes was 23 in endometrium cancer, and 26 and 28 in ovarian and cervical cancers, respectively. Additionally, the mean count of dissected pelvic lymph nodes was 26 in the loupe-negative and 20 in the loupe-positive groups. According to previous reports, the optimal count of para-aortic lymph nodes is 10 for gynecologic cancers. A study found a positive correlation between the count of dissected para-aortic lymph nodes and the formation of chylous ascites^(14)^. In our study, the mean count of dissected para-aortic lymph nodes was 12 in the loupe-negative group and 13.5 in the loupe-positive group (p=0.93). Nevertheless, we found no significant relationship between the count of dissected para-aortic lymph nodes and the amount of lymphatic drainage.

### Study Limitations

We analyzed whether using loupes decreased the most common complications of lymph node dissection; prolonged lymphatic discharge, prolonged drainage use, and lymphocele formation and hemorrhage. More studies are needed to evaluate oncologic outcomes. The main limitation of our study was the number of patients included to evaluate lymphocele formation; however, there was an enormous decrease in total drainage volume. This large difference decreased the number of patients required to randomize for a sufficient power.

## Conclusion

In gynecological cancer surgeries, using loupes can decrease lymphocele formation and the amount of lymphatic drainage. Therefore, drains can be removed earlier as a part of enhanced recovery after surgery. Moreover, magnification can be beneficial in order to correctly apply clips to target vessels and decrease the number of clips required. This may reduce complications as well as the total cost of surgery. Although loupe magnification is used in many surgical practices, it is still yet not common in gynecologic oncologic procedures. More studies should be undertaken the evaluate the results of gynecologic surgery performed with loupes.

## Figures and Tables

**Table 1:**
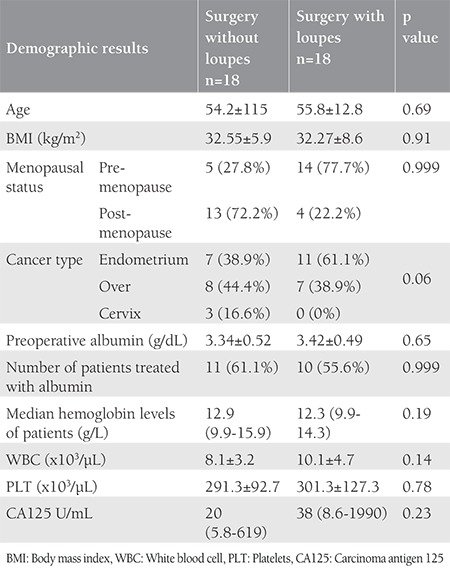
Demographic results and laboratory findings of the groups

**Table 2:**
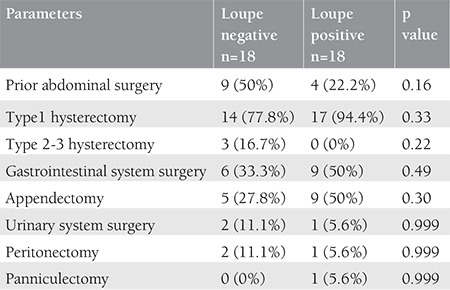
Evaluation of surgical procedures between the two groups

**Table 3:**
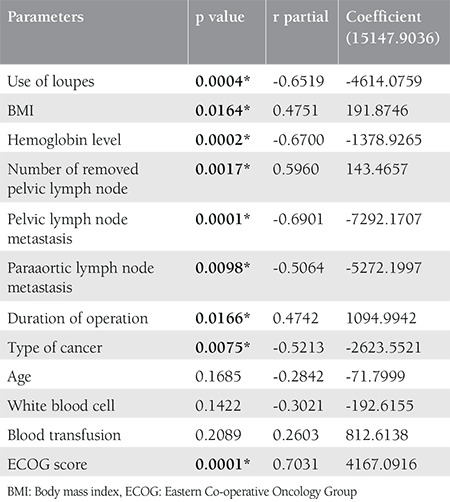
The results of multiple regression analysis
